# Immunogenic cell death-related long noncoding RNA influences immunotherapy against lung adenocarcinoma

**DOI:** 10.32604/or.2023.029287

**Published:** 2023-07-21

**Authors:** DONGJIE SUN, CHI ZHANG

**Affiliations:** 1 Department of Translational Medicine, The First Hospital of Jilin University, Changchun, China; 2 College of Basic Medical Sciences, Jilin University, Changchun, China; 3 Department of Anesthesiology, The First Hospital of Jilin University, Changchun, China

**Keywords:** Lung adenocarcinoma, Immunogenic cell death, Prognostic model, Bioinformatics, Tumor infiltration

## Abstract

Lung adenocarcinoma (LUAD) is the leading cause of cancer-related deaths, accounting for over a million deaths worldwide annually. Immunogenic cell death (ICD) elicits an adaptive immune response. However, the role of ICD-related long noncoding RNAs (lncRNAs) in LUAD is unknown. In this study, we investigated the characteristics of the tumor microenvironment in LUAD, the prognostic significance of ICD-related lncRNAs, and the half-maximal inhibitory concentration (IC50) of possible chemotherapeutic drugs. We sorted prognostic lncRNAs using univariate Cox regression and constructed a risk signature based on them. We then confirmed the model’s accuracy and generated a nomogram. Additionally, we performed immune microenvironment analysis, somatic mutation calculation, Tumor Immune Dysfunction and Exclusion (TIDE) analysis, and anticancer pharmaceutical IC50 prediction. Least absolute shrinkage and selection operator Cox regression identified 27 prognostic lncRNAs related to ICD, and a unique risk signature using 10 ICD-related lncRNAs was constructed. The risk score was confirmed to be a reliable predictor of survival, with the highest c-index score. The signature had a remarkable predictive performance with clinical applicability and could accurately predict the overall survival in LUAD. Furthermore, the lncRNA signature was closely associated with immunocyte invasion. We also analyzed the correlation between the risk score, tumor-infiltrating immune cells, and prognosis and identified high immune and ESTIMATE scores in low-risk patients. Moreover, we observed elevated checkpoint gene expression and low TIDE scores in high-risk patients, indicating a good immunotherapy response. Finally, high-risk patients were shown to be susceptible to anticancer medications. Therefore, our unique risk signature comprising 10 ICD-related lncRNAs was demonstrated to indicate the characteristics of the tumor-immune microenvironment in LUAD, predict patients’ overall survival, and guide individualized treatment.

## Introduction

Lung adenocarcinoma (LUAD) accounts for approximately 40% of all lung cancer cases and causes 1.76 million deaths worldwide annually with a less than 20% 5-year survival rate [1,2]. Despite advancements in cancer therapy, LUAD survival remains poor because of a lack of early prognostic indications. Therefore, it is vital to develop a simple and efficient prognostic model to assess a patient’s prognosis and guide personalized therapies.

Immunogenic cell death (ICD) is a type of antitumor immunity that alters the immunological microenvironments of tumors by emitting warning signals, which can benefit immunotherapy [3,4]. A tyrosine kinase inhibitor that induces ICD has shown excellent antitumor activity when combined with non-ICD-inducing drugs such as cisplatin and crizotinib. Preclinical evidence suggests that cytotoxic agents that induce ICD (such as oxaliplatin and cyclophosphamide) can improve immunotherapy for non-small cell lung cancer (NSCLC) [5]. ICD enhances the antitumor immune response by transforming dying malignant cells into medicinal immunizations. Tumors with a higher propensity for ICD may provoke stronger antitumor immune responses, which can help suppress tumor growth. Therefore, further research is essential to investigate the potential of ICD-related anticancer therapy for LUAD.

Long noncoding RNAs (lncRNAs) have gained attention as a potentially crucial component of biological control [6]. For instance, the lncRNA *TUC338* has been shown to promote lung cancer invasion by activating the mitogen-activated protein kinase (MAPK) pathway [7]. Additionally, *KCNQ1OT1* is highly expressed in NSCLC and indicates a poor patient prognosis, suggesting that it may serve as a molecular marker for the prognosis of NSCLC [8]. Several lncRNAs, including ferroptosis-related [9,10], m6A-related [11], and pyroptosis-related lncRNAs [12], have been implicated in the pathogenesis and prognosis of LUAD patients. However, it remains unclear how ICD-associated lncRNAs affect LUAD.

This study aimed to investigate the role of ICD-associated lncRNAs in LUAD; a 10 ICD-associated lncRNA risk signature was discovered that could predict outcome and describe the tumor-immune milieu. Using this signature, a nomogram was developed to improve prognostic accuracy. Our risk model performed better than others in terms of predicting overall survival (OS) and drug sensitivity. These findings elucidate the function of ICD in LUAD and provide the basis for developing new targeted anticancer drugs.

## Materials and Methods

### Raw data processing

RNA-seq profiles (FPKM) were obtained from The Cancer Genome Atlas (TCGA) database (https://tcga-data.nci.nih.gov/tcga/) for 539 LUAD cancer samples and 59 noncancerous samples. Clinical data were retrieved and updated on May 31, 2022. Patients whose clinical information was unavailable were excluded from further analysis. The 34 ICD genes used in earlier investigations were also obtained [13].

### Selection of ICD‑related lncRNAs

To identify ICD-related lncRNAs, the mRNA expression of the 34 ICD-related genes was retrieved, and Pearson’s correlation test was performed to examine the correlation between ICD-related genes and lncRNA expression profiles. ICD‑related lncRNAs were identified as those with R > 0.4 and *p* < 0.001.

### Construction of an ICD-related lncRNA prognostic model

Survival-associated lncRNAs were filtered using univariate Cox regression with a *p*-value < 0.01. Least absolute shrinkage and selection operator (LASSO)-Cox regression was then performed to construct a risk prediction model to prevent overfitting. The risk scores were calculated using the following formula:

$$risk\;score=\overset{n}{\underset{i=1}{\sum }}\left(coef_{i}*expr_{i}\right),$$

where $coef_{i}$ represents each lncRNA’s coefficients and $expr_{i}$ represents the expression level.

### Risk signature validation and nomogram establishment

The risk model’s validity and dependability were assessed using Kaplan–Meier survival analysis. Individuals were grouped into high- or low-risk groups based on median risk scores, each comprising 50% of the population. The “survminer” package was used to generate survival curves. Cox regression was used to examine clinical data and risk scores to forecast OS independence. The concordance index (C-index) and time-dependent receiver operating characteristic (ROC) curves were used to determine the predictive capacity of risk scores using the “survcomp” and “survivalROC” packages. The “rms” package was used to create a nomogram with correction curves.

### Immune infiltration analysis

The CIBERSORT method was applied to analyze the transcriptomic data of the LUAD cohort to illustrate the proportion of 22 tumor-infiltrating immune cells (TICs). Next, the correlation between risk scores and TICs was analyzed. The stromal score and immune score were calculated using the “ESTIMATE” package.

### Tumor immune dysfunction and exclusion (TIDE) analysis

TIDE scores were used to predict a patient’s responsiveness to immunotherapy (http://tide.dfci.harvard.edu/) [14]. An increased TIDE score suggested an elevated chance of tumor escape from the immune response.

### Mutation analysis

The “maftools” package was used to create somatic mutation charts for high- and low-risk groups. Furthermore, we generated survival curves across subgroups based on the median mutation value and LUAD patients’ risk scores.

### Functional enrichment analysis

Differentially expressed genes (DEGs) between risk groups were identified (adj. *p* < 0.05, |log_2_FC| > 1). Next, functional enrichment was applied using Kyoto Encyclopedia of Genes and Genomes (KEGG) and Gene Ontology (GO) analysis with the “clusterProfiler” package. The “GSEA” package was used to compare gene set alteration across risk groups (c2.cp.kegg.v7.4.symbols.gmt).

### Prediction of drug susceptibility

The half-maximal inhibitory concentration of antitumor drugs was predicted in risk groups using the “pRRophetic” package.

### Real-time quantitative polymerase chain reaction (RT-qPCR) analysis

We detected the mRNA expression level of *LY86-AS1* in BEAS-2B, A549, and PC-9 cell lines. Total RNA (1 μg) was isolated using the TRIzol reagent (Invitrogen, USA), and first-strand complementary DNA was synthesized using SuperScript III Reverse Transcriptase (Invitrogen, USA) and oligo-dT (Promega, USA), according to the manufacturer’s instructions. qPCR was performed using SYBR green (Sigma, USA). The 2^−ΔΔCT^ calculation method was performed. Glyceraldehyde 3-phosphate dehydrogenase (GAPDH) was employed as an endogenous control. The primer sequences were as follows: *LY86-AS1*: forward 5′-TCAATTCAGATTTGGAGGGC-3′, reverse 5′-GTTGAAGTTCATCTCTTCAACC-3′; and GAPDH: forward 5′-TCAAGATCATCAGCAATGCC-3′, reverse 5′-CGATACCAAAGTTGTCATGGA-3′.

### Statistical analysis

All statistical computations were performed using the R program (version 4.1.3). For analysis of categorical data, the Chi-square test was employed. The association between risk scores and drug sensitivity was assessed using Spearman’s correlation analysis. A two-sided *p* < 0.05 was considered statistically significant.

## Results

### Identification of ICD-related lncRNAs in LUAD

**Figure 1:**
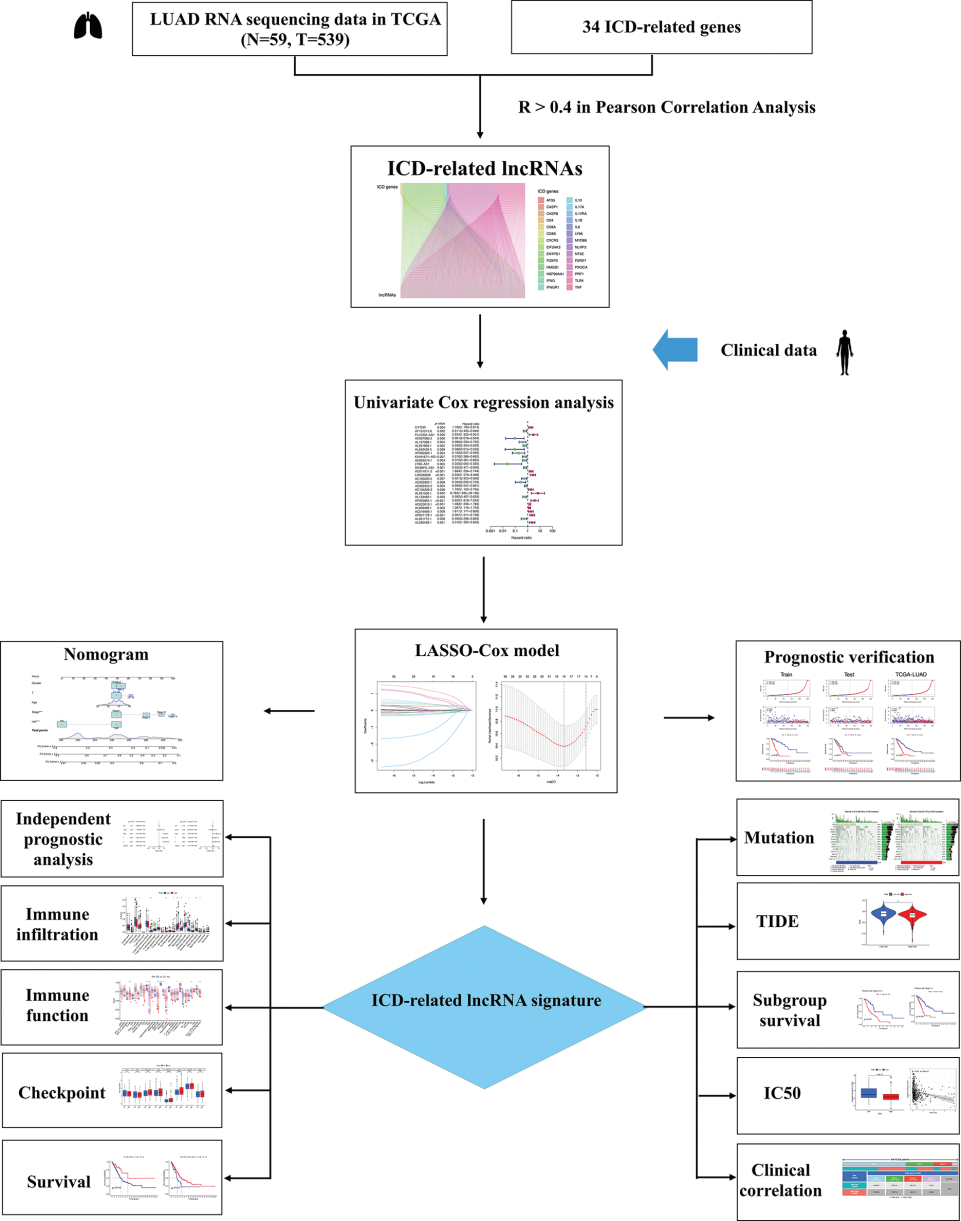
Flow chart of the study.

**Figure 2:**
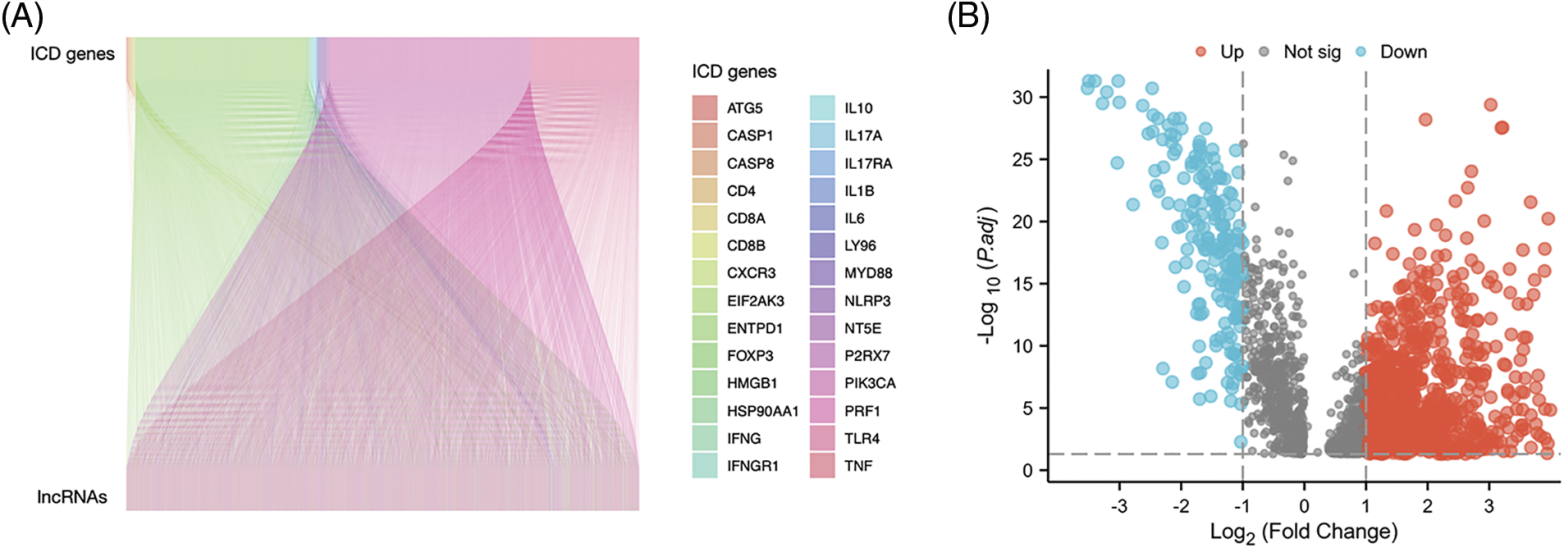
Immunogenic cell death (ICD)–related long noncoding RNA (lncRNA) identification. (A) Correlation between ICD genes and associated lncRNAs. (B) Heatmap of 2,619 ICD-related lncRNAs.

Fig. 1 presents the study workflow. In total, 16,876 lncRNAs and 19,938 mRNAs were identified from the LUAD cohort. The 34 ICD genes and correlated lncRNAs were analyzed (Fig. 2A). In total, 2,619 ICD-related lncRNAs were identified, of which the expressions of 890 were upregulated, and the expressions of 193 lncRNAs were downregulated (|log_2_(fold change)| > 1 and adjusted *p* < 0.05) (Fig. 2B).

### Establishing a risk model for LUAD patients using 10 ICD-related lncRNAs

**Figure 3:**
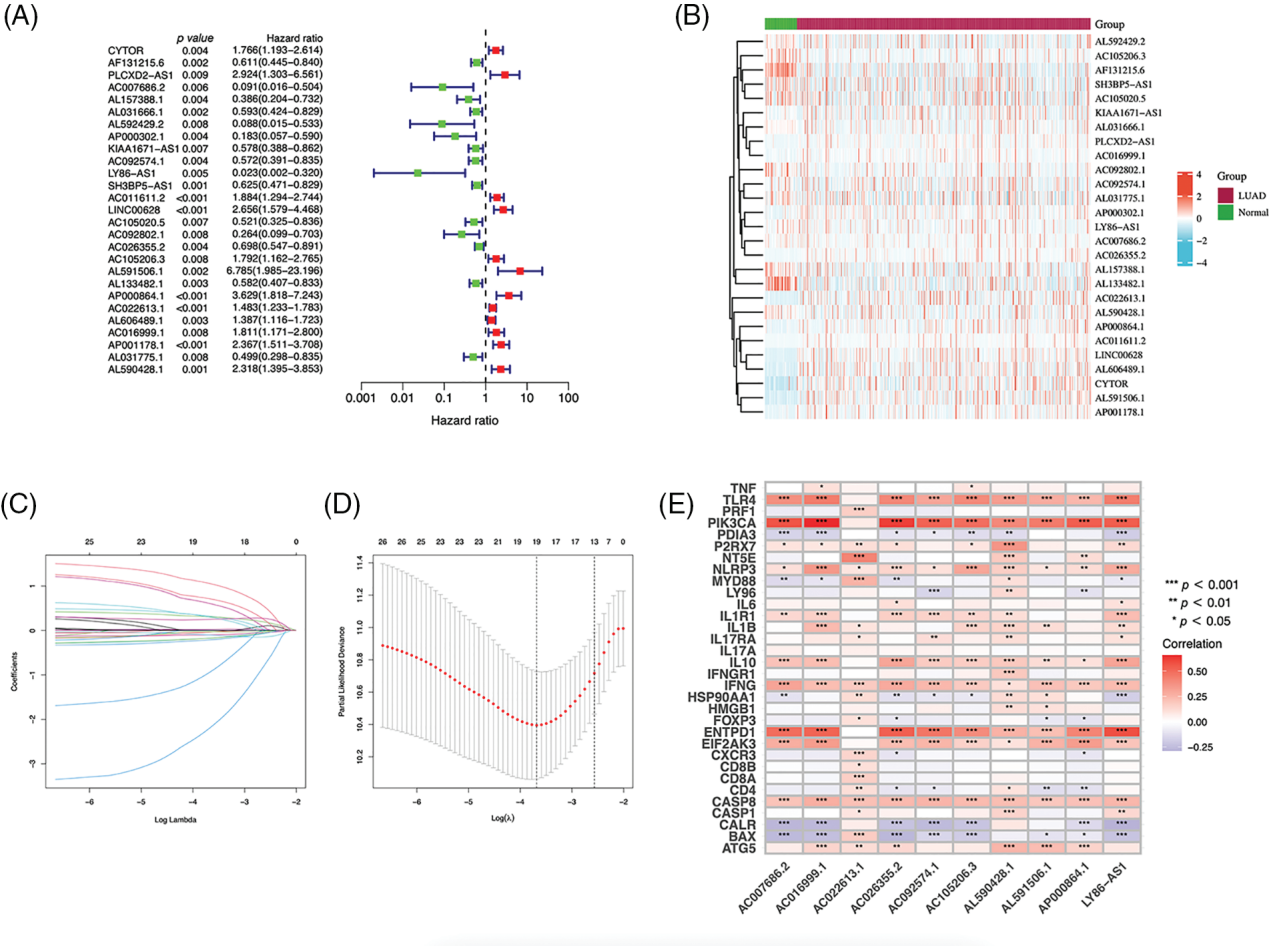
Immunogenic cell death (ICD)–related long noncoding RNA (lncRNA) model construction. (A) Univariate Cox regression analyses of ICD-related lncRNAs in lung adenocarcinoma (LUAD). (B) Prognostic lncRNAs in LUAD. (C) Least absolute shrinkage and selection operator (LASSO) coefficient distribution. (D) Optimized lambda determined using the LASSO regression model. (E) The correlation of prognostic ICD-related lncRNAs and ICD genes. *p < 0.05, **p < 0.01, ***p < 0.001.

**Figure 4:**
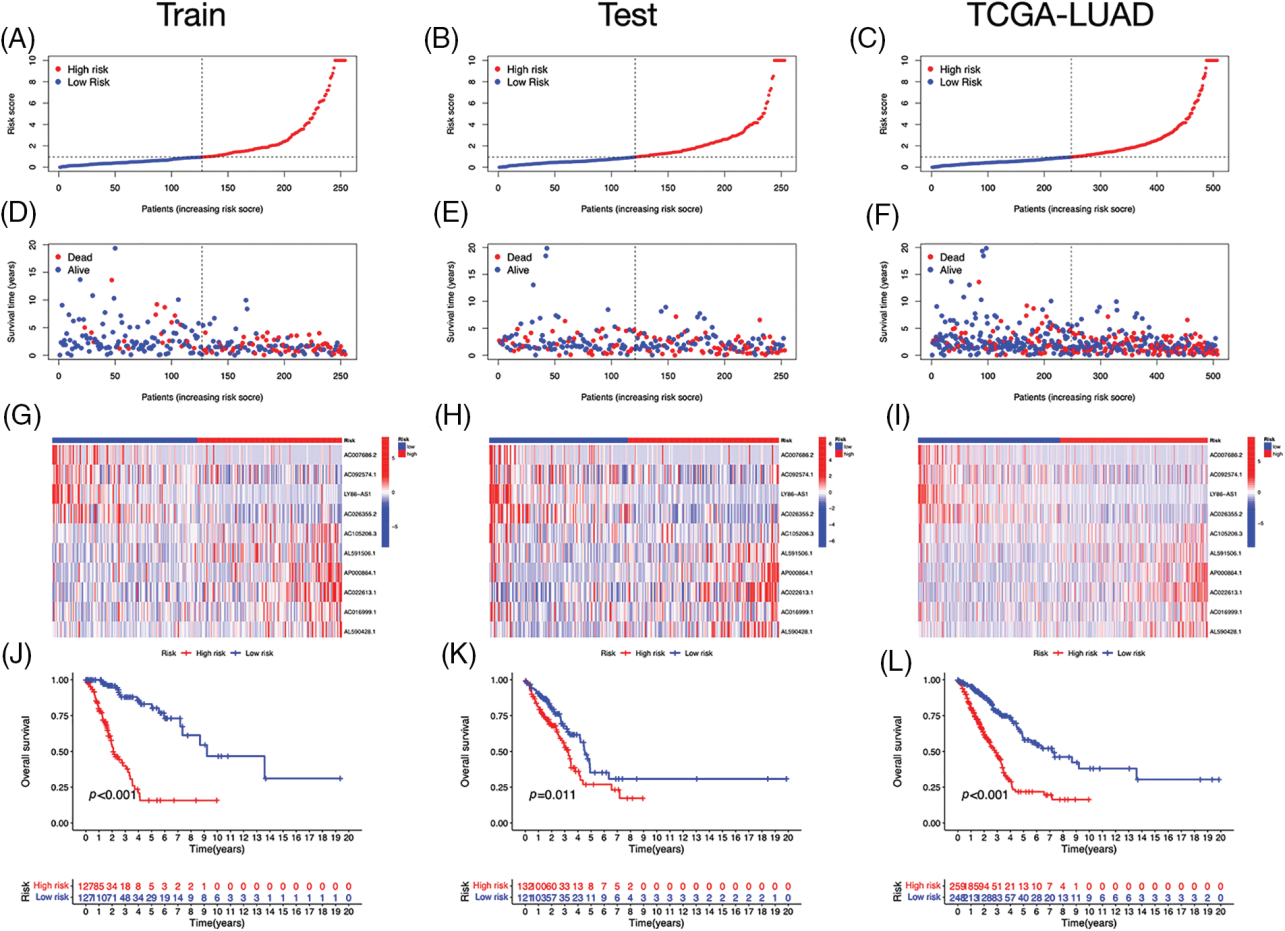
Prognostic value of the risk signature. (A–C) The distribution of risk scores in the training, test, and The Cancer Genome Atlas (TCGA)-lung adenocarcinoma (LUAD) cohorts. (D–F) Survival status in the training, test, and TCGA-LUAD cohorts. (G–I) Heatmaps of the expression of ICD-related lncRNAs in the training, test, and TCGA-LUAD cohorts. (J–L) Kaplan–Meier survival curves of OS in the training, test, and TCGA-LUAD cohorts.

Using univariate Cox regression, we identified 27 lncRNAs associated with ICD that were substantially related to OS (Figs. 3A and 3B). LASSO regression analysis was performed to avoid overfitting (Figs. 3C and 3D). Thereafter, multivariate Cox regression was used for hub lncRNA selection and coefficient calculation of the risk model. Finally, 10 prognostic lncRNAs associated with ICD genes were screened out. Fig. 3E shows the association between the 10 lncRNAs and ICD genes. Each sample’s risk score was calculated as follows: Risk Score = AC007686.2 × (−2.0061) + AC092574.1 × (−0.3296) + LY86-AS1 × (−4.0825) + AC026355.2 × (−0.3458) + AC105206.3 × (0.4674) + AL591506.1 × (1.2057) + AP000864.1 × (1.3663) + AC022613.1 × (0.3891) + AC016999.1 × (0.6251) + AL590428.1 × (0.9760). Patients in TCGA-LUAD cohort were randomized into high- or low-risk groups based on the median risk score. Subsequently, OS in the training, test, and the whole TCGA-LUAD cohorts were compared (Figs. 4A–4C). A higher mortality rate was observed in the high-risk group (Figs. 4D–4F). Figs. 4G–4I show the expression of the 10 lncRNAs in each cohort. Patients in the high-risk group across the three cohorts showed poor prognoses (Figs. 4J–4L). Detailed clinical data are shown in Table 1, which were not significantly biased between the training and test sets.

**Table 1:** Clinicopathologic features of LUAD patients

Features	Total	Test	Train	*p* value
Age				
<=65	239 (47.14%)	114 (45.06%)	125 (49.21%)	0.3928
>65	258 (50.89%)	134 (52.96%)	124 (48.82%)	
Unknow	10 (1.97%)	5 (1.98%)	5 (1.97%)	
Gender				
FEMALE	272 (53.65%)	140 (55.34%)	132 (51.97%)	0.5021
MALE	235 (46.35%)	113 (44.66%)	122 (48.03%)	
Stage				
Stage I	272 (53.65%)	131 (51.78%)	141 (55.51%)	0.7392
Stage II	120 (23.67%)	62 (24.51%)	58 (22.83%)	
Stage III	81 (15.98%)	44 (17.39%)	37 (14.57%)	
Stage IV	26 (5.13%)	12 (4.74%)	14 (5.51%)	
Unknow	8 (1.58%)	4 (1.58%)	4 (1.57%)	
T				
T1	169 (33.33%)	79 (31.23%)	90 (35.43%)	0.3893
T2	271 (53.45%)	137 (54.15%)	134 (52.76%)	
T3	45 (8.88%)	27 (10.67%)	18 (7.09%)	
T4	19 (3.75%)	8 (3.16%)	11 (4.33%)	
Unknow	3 (0.59%)	2 (0.79%)	1 (0.39%)	
M				
M0	338 (66.67%)	172 (67.98%)	166 (65.35%)	0.9431
M1	25 (4.93%)	12 (4.74%)	13 (5.12%)	
Unknow	144 (28.4%)	69 (27.27%)	75 (29.53%)	
N				
N0	327 (64.5%)	161 (63.64%)	166 (65.35%)	0.5133
N1	95 (18.74%)	49 (19.37%)	46 (18.11%)	
N2	71 (14%)	37 (14.62%)	34 (13.39%)	
N3	2 (0.39%)	0 (0%)	2 (0.79%)	
Unknow	12 (2.37%)	6 (2.37%)	6 (2.36%)	

### Relationships between lncRNAs associated with ICD and clinical pathological variables

**Figure 5:**
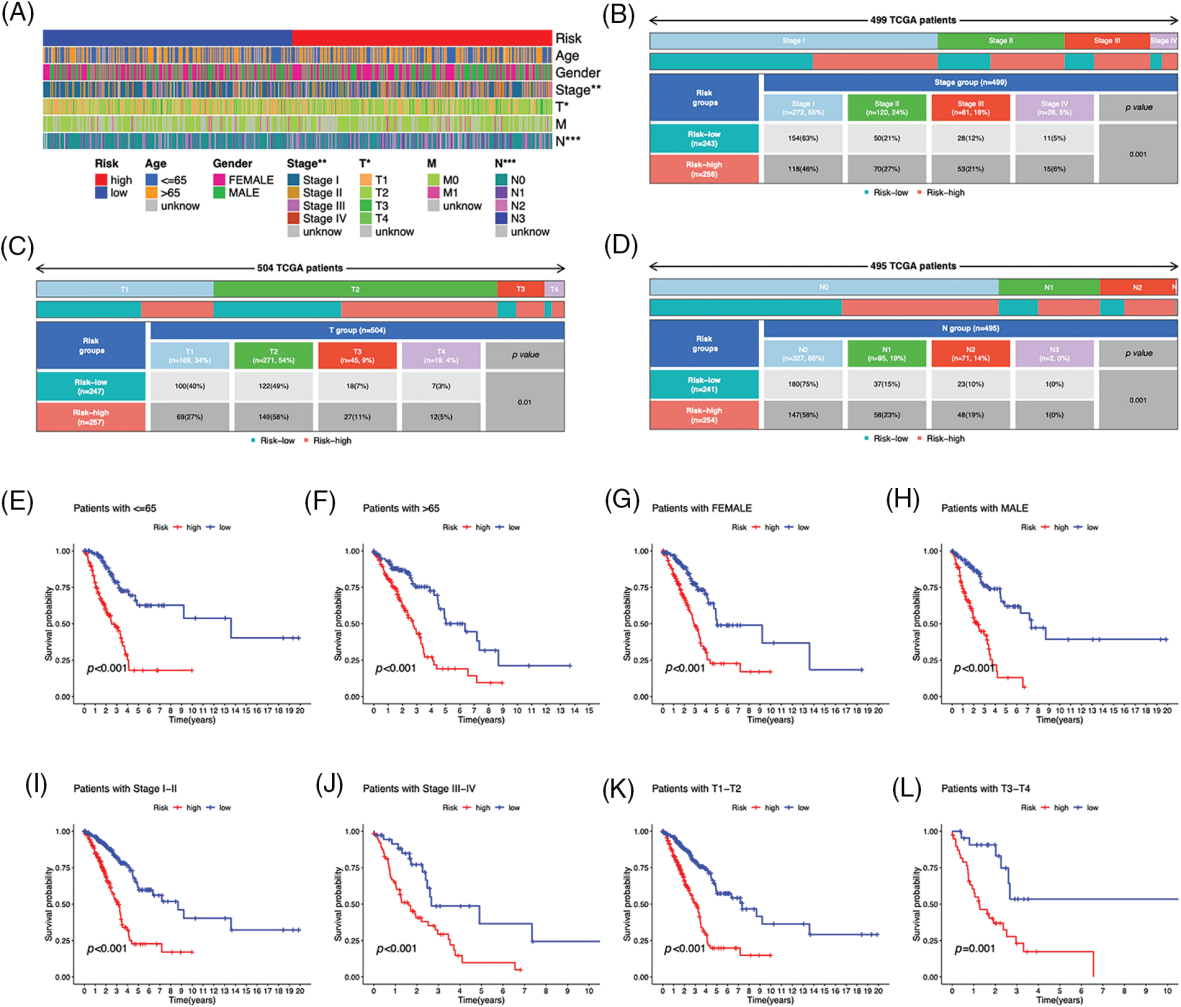
Clinicopathological features in the risk model. (A) Distribution of each sample’s clinicopathological features and associated risk scores. (B–D) Distribution of patients in different risk groups. (E–L) OS curves for subgroups analysis. *p < 0.05, **p < 0.01, ***p < 0.001.

Based on the heatmap, the tumor, T, and N stages were significantly different between risk groups, whereas other variables such as age, gender, and M stage did not differ substantially (Fig. 5A). The distribution of observations in the two groups according to the T stage and N stage is shown in Figs. 5B–5D. To determine the risk scores in subgroups, we conducted a subgroup survival analysis by categorizing them based on age (≤65 or >65 years), gender (male or female), stages (I–II or III–IV), and T stage (T1–2 or T3–4). Figs. 5E–5L demonstrate that individuals classified as low-risk had higher OS rates than those classified as high-risk, across different subgroups, including sex, age groups (≤65 or >65 years), T1–T2 or T3–T4 tumor stages, and stages I–II or III–IV.

### Nomogram construction

**Figure 6:**
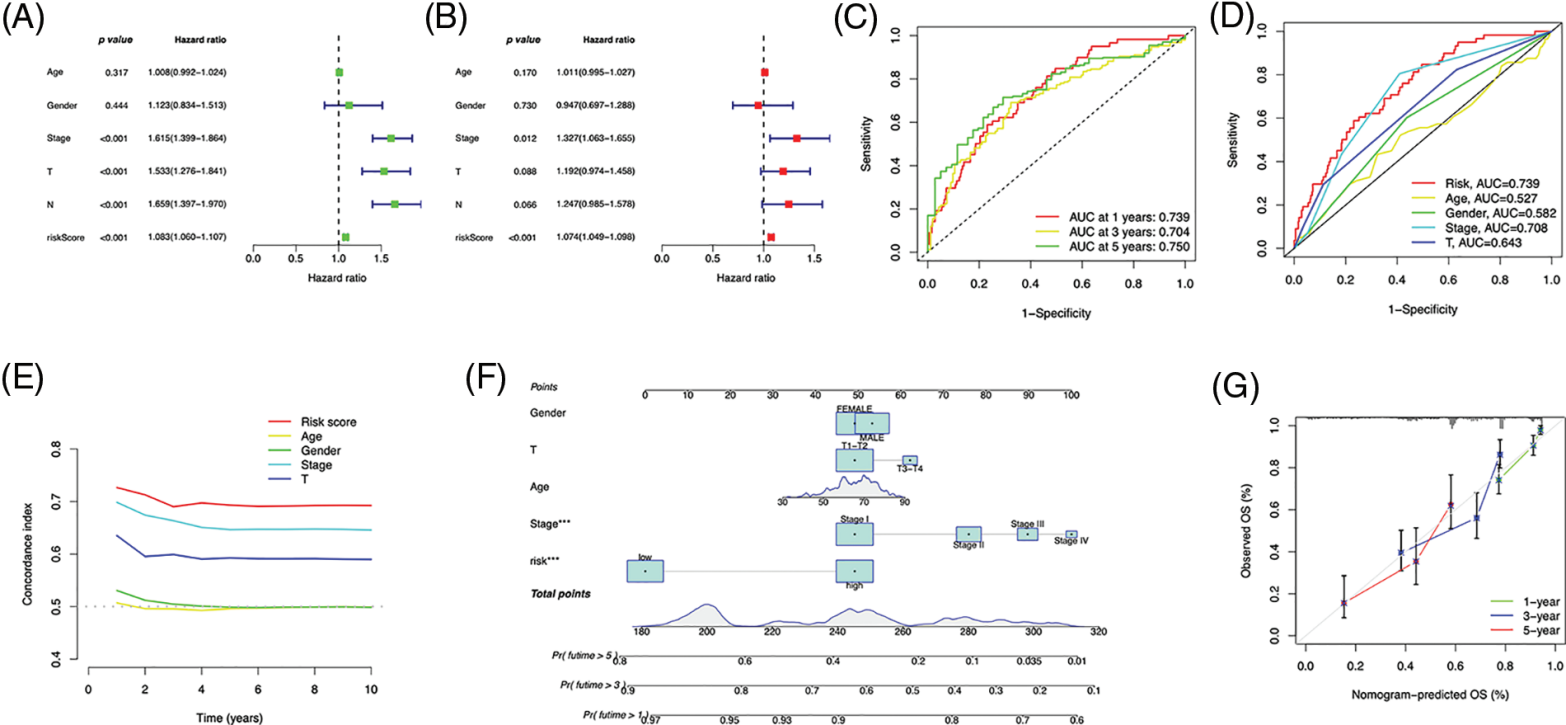
Prognostic value of the risk signature. (A) Univariate and (B) multivariate Cox regression for prognosis analysis. (C) Time-dependent ROC curve prediction. (D) Predictive accuracy analysis. (E) C-index analysis. (F) Nomogram combining the risk score and clinicopathological variables for OS prediction. (G) Calibration curves for survival prediction.

The independent prognostic analysis, utilizing univariate and multivariate analysis, showed that the risk score could predict OS in LUAD patients independently (Figs. 6A and 6B). The area under the curve (AUC) reached 0.739, 0.704, and 0.750 for one, three, and five years, respectively (Fig. 6C), and demonstrated a higher predictive power than that of other clinical parameters (Figs. 6D and 6E). We developed a nomogram, including the risk score and clinical characteristics, to predict OS in LUAD patients (Fig. 6F). The calibration curves exhibited a strong match between predictions and observations (Fig. 6G).

### Immune features of the risk model

**Figure 7:**
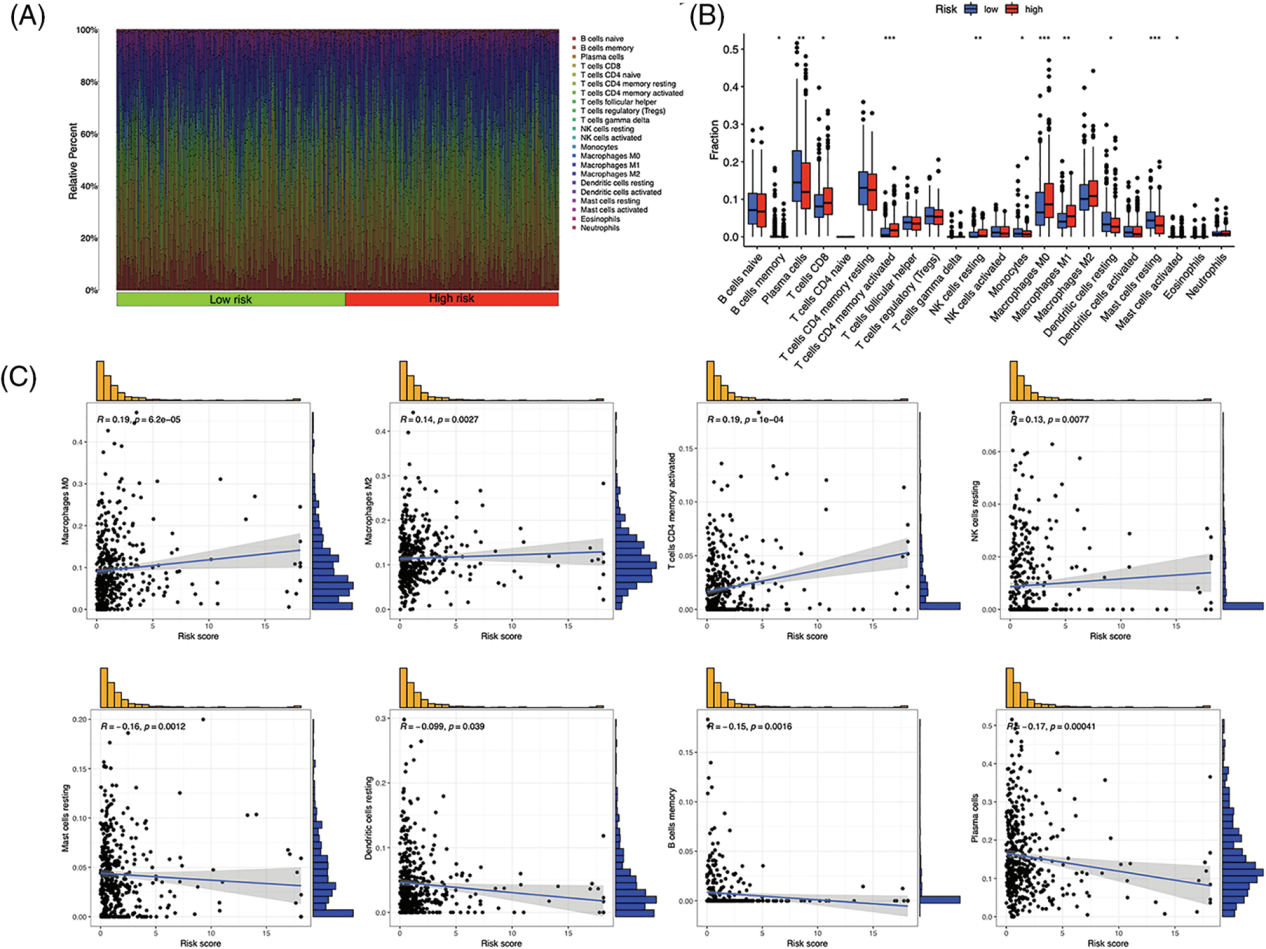
Tumor-infiltrating immune cells (TICs) of the risk signature. (A) Distribution of TICs in high- and low-risk groups. (B) Different TICs across risk groups. (C) Correlation between lymphocytes and risk scores. *p < 0.05, **p < 0.01, ***p < 0.001.

**Figure 8:**
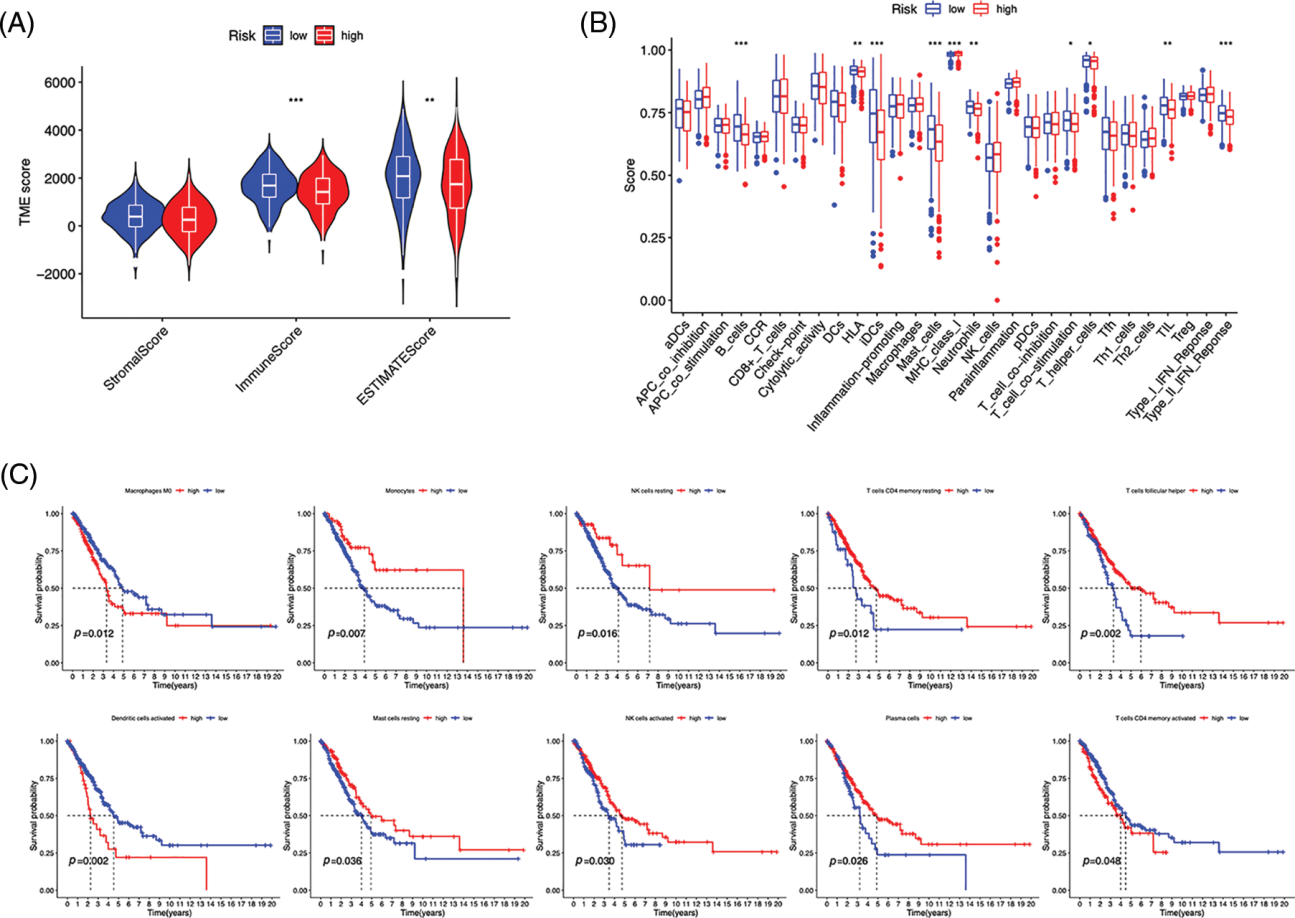
Immune profiles of risk groups. (A) Tumor microenvironment scores of risk groups. (B) Immune cells and function enrichment. (C) Survival probability of patients with high and low immune cell expression. *p < 0.05, **p < 0.01, ***p < 0.001.

Immunocytes were found to differ between risk groups (Fig. 7A), and a strong association between immune cells was discovered (Fig. 7B). The low-risk group had more resting dendritic cells (DCs), activated CD4+ memory T cells, memory B cells, plasma cells, and resting mast cells. High numbers of CD8+ T cells, resting natural killer (NK) cells, and M0 and M1 macrophages were observed in the high-risk group. Activated CD4+ T cells, M0 and M2 phenotype macrophages, plasma cells, and resting NK cells were negatively correlated with risk scores. In contrast, resting DCs, plasma cells, memory B cells, and resting mast cells were positively correlated with risk scores (Fig. 7C). The correlations between ICD genes and risk scores are shown in Suppl. Fig. 1. Moreover, the high-risk group had lower immune and overall ESTIMATE scores, suggesting a higher tumor proportion (Fig. 8A). ssGSEA revealed that the low-risk group exhibited a more significant infiltration of immunocytes, including Th2 cells, DCs, T helper cells, neutrophils, mast cells, and B cells (Fig. 8B). Furthermore, Fig. 8C shows the correlation between immune cell proportion and survival probability. These findings suggest that ICD-related risk scores could distinguish between distinct aspects of TICs in LUAD, with the low-risk group showing increased immunocyte infiltration.

### Functional enrichment and genetic variations in risk groups

**Figure 9:**
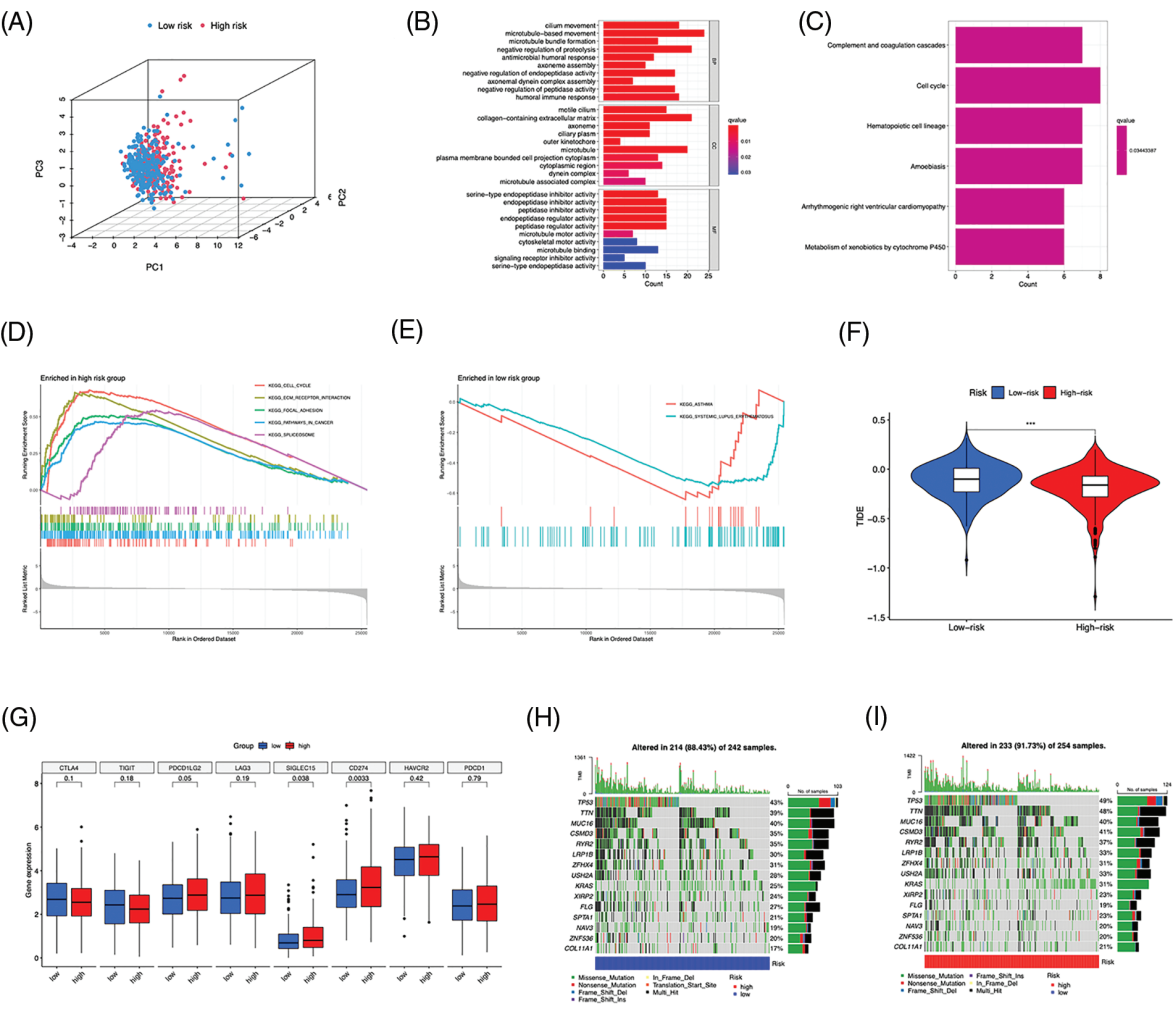
Functional enrichment and genomic alteration analysis. (A) PCA based on the 10 risk long noncoding RNAs (lncRNAs). GO (B) and KEGG (C) enrichment of DEGs. Genes enriched in the high- (D) and low-risk groups (E). (F) TIDE scores analysis. (G) Differential expression of multiple immune checkpoints. (H, I) Oncoprint of the most mutated genes. ***p < 0.001.

LUAD patients were divided into two groups using principal component analysis (PCA) based on risk scores (Fig. 9A). In total, 481 DEGs, primarily enriched in the humoral immune response and cell cycle, were identified (Figs. 9B and 9C). Furthermore, GSEA revealed that DEGs in the high-risk group were enriched in the cell cycle, extracellular matrix (ECM)-receptor interaction, and focal adhesion pathways (Fig. 9D). The low-risk group was primarily associated with asthma and systemic lupus erythematosus (Fig. 9E). To evaluate the risk signature’s ability to predict the clinical effectiveness of immunotherapy, we used TIDE analysis. Our findings showed that the TIDE score of the high-risk group was reduced (Fig. 9F), indicating an increased likelihood of tumor escape from immunotherapy in individuals with lower risk. Next, we examined the expression of immunological checkpoint genes and discovered that *PDCD1LG2* (PD-L2), *SIGLEC15*, and *CD274* were more highly expressed in high-risk individuals (Fig. 9G). Finally, we identified somatic mutations; Figs. 9H and 9I show the most frequently mutated genes, with *TP53* ranking first.

### Chemotherapeutics prediction

**Figure 10:**
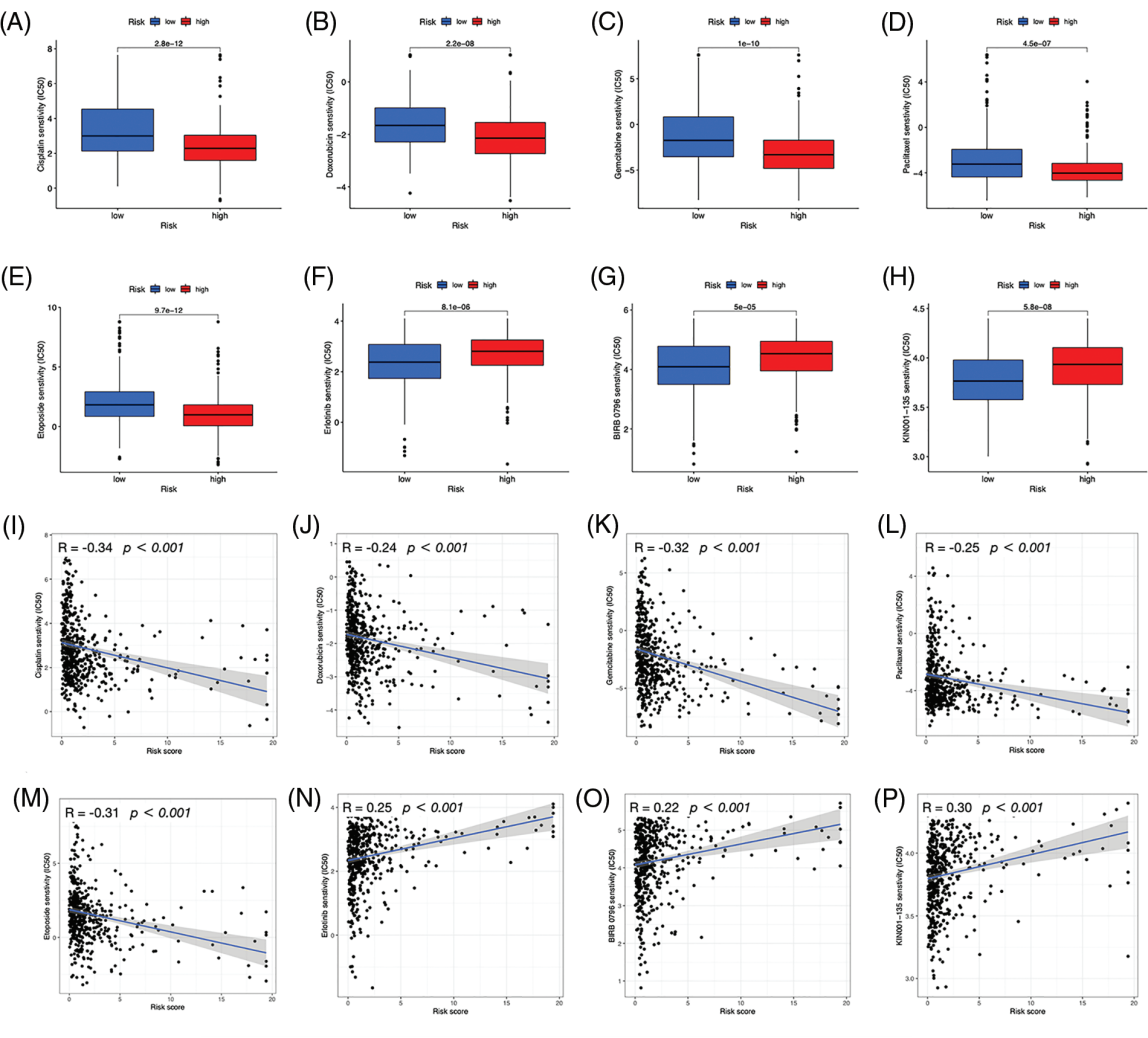
Prediction of drug susceptibility. Sensitive drugs for the high-risk group (A–E) and low-risk group (F–H). (I–P) Correlation between risk scores and drugs.

We investigated the potential therapeutic application of the two risk groups by analyzing medication sensitivity. The findings demonstrated that doxorubicin, gemcitabine, paclitaxel, cisplatin, and etoposide had potential effects on high-risk individuals (Figs. 10A–10E). Individuals at low risk showed increased sensitivity to erlotinib (Fig. 10F), BIRB-0796 (p38 MAPK inhibitors) (Fig. 10G), and KIN001-135 (Fig. 10H). The correlation between risk scores and sensitive drugs was also analyzed (Figs. 10I–10P). However, BIRB-0796 is currently only used for scientific research, and KIN001-135 has not yet been applied in public research. In clinical practice, these two sensitive drugs have not been administered to a group of low-risk individuals but may have therapeutic potential.

### Comprehensive analysis of LY86-AS1 and validation

**Figure 11:**
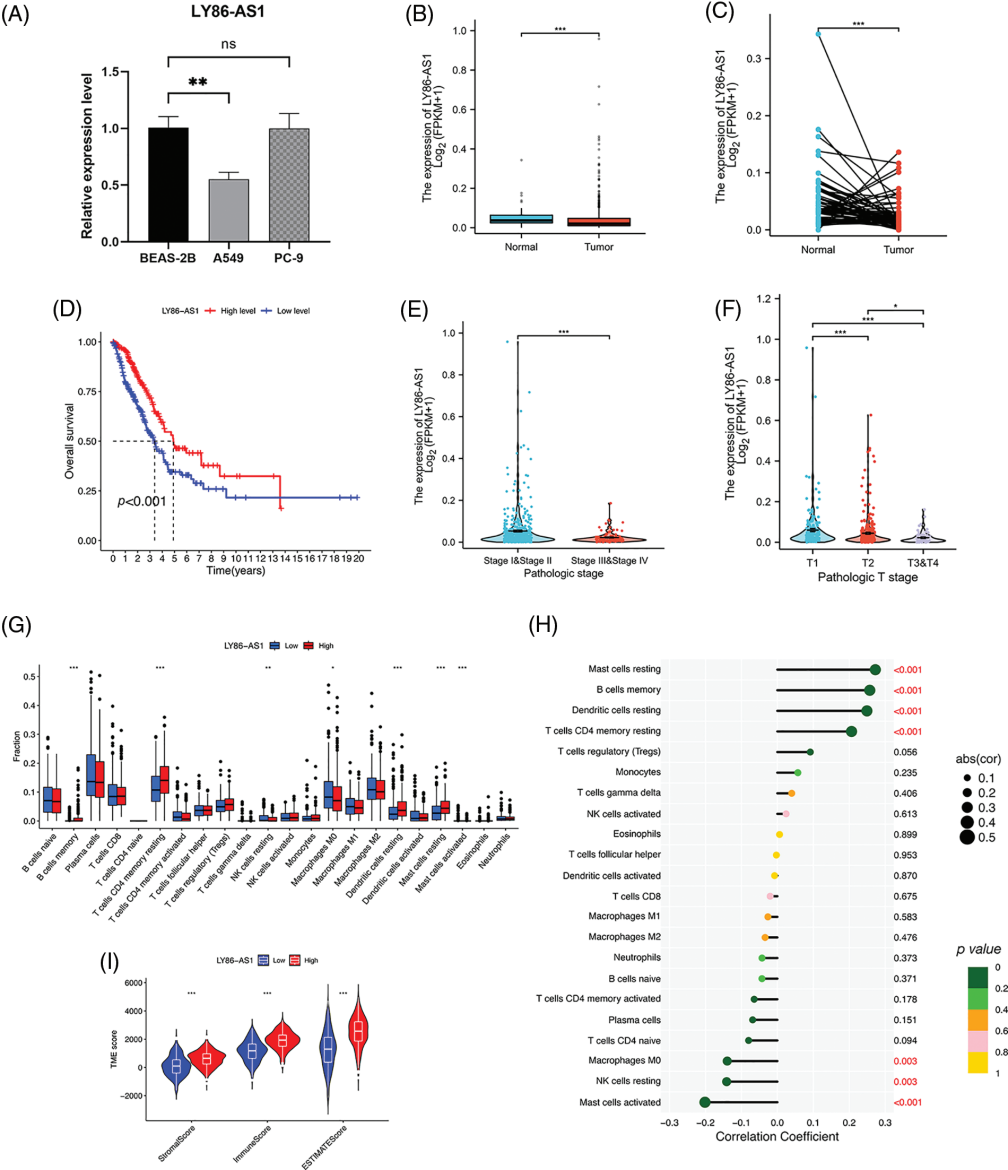
Comprehensive analysis of LY86-AS1. (A) RT-qPCR analysis of LY86-AS1 in the lung adenocarcinoma (LUAD) cell lines and control group. The expression level of LY86-AS1 in unpaired (B) and paired patients (C) in TCGA-LUAD cohort. (D) OS curves of patients in the high- and low-LY86-AS1 expression groups. Correlation between the expression level of LY86-AS1 and tumor (E) and T (F) stages. (G) Distribution of tumor-infiltrating immune cells (TICs) in high- and low-LY86-AS1 expression groups. (H) Correlation between the expression level of LY86-AS1 and TICs. (I) Tumor microenvironment scores of high- and low-LY86-AS1 expression groups. *p < 0.05, **p < 0.01, ***p < 0.001.

We validated the mRNA expression level of LY86-AS1 in two LUAD cell lines and the control group. The results showed that LY86-AS1 expression was significantly downregulated in A549 cells compared to that in BEAS-2B cells; however, no changes in LY86-AS1 expression were seen in PC-9 cells (Fig. 11A). Additionally, we investigated the expression level of LY86-AS1 in the LUAD cohort and found it to be downregulated in both unpaired (Fig. 11B) and paired patients (Fig. 11C). Patients with high levels of LY86-AS1 had better OS than those with low levels (Fig. 11D), and patients with high tumor and T stages had low LY86-AS1 expression (Figs. 11E and 11F). The distribution of TICs differed between the high- and low-LY86-AS1 expression groups (Fig. 11G). The proportions of memory B cells, resting memory CD4 T cells, resting DCs, and resting mast cells were low in the low-LY86-AS1 expression group, whereas the proportion of M0 macrophages was high in the low-LY86-AS1 expression group. The correlation between the expression level of LY86-AS1 and TICs was demonstrated in Fig. 11H. Tumor microenvironment (TME) score analysis showed that the high-LY86-AS1 expression group had high immune scores (Fig. 11I).

## Discussion

To treat LUAD, the combination of immunogenic therapy and innovative immunotherapeutic regimens has shown considerable potential [4]. However, access to innovative treatments for this aggressive cancer is limited, partly because of immunological resistance. In recent decades, the identification of ICD has elucidated the pertinent relationship between dying malignant cells and adaptive immunocytes in cancer treatment [15–17]. ICD modifies the tumor immunological microenvironment by emitting danger signals or DAMPs, thus potentially benefiting immunotherapy. Therefore, ICD-related biomarkers may aid in identifying LUAD patients who could benefit from antitumor therapy.

lncRNAs have been influential in supporting many biological functions in LUAD etiology, and LUAD development and progression are connected to lncRNA anomalies [18]. However, research on ICD-related lncRNAs for predicting LUAD survival is limited. In this study, we demonstrate that ICD-related lncRNAs affect LUAD’s prognosis and immunology. Furthermore, we created a prognostic risk signature with 10 ICD-associated lncRNAs and classified LUAD patients as high-risk or low-risk accordingly. This risk signature demonstrated a high predictive power for OS and could also reflect the immunocytes infiltration and drug sensitivity of the risk groups, which might contribute to LUAD treatment.

This study identified 10 ICD-related lncRNAs that are associated with LUAD prognosis, including AC007686.2, AC092574.1, LY86-AS1, AC026355.2, AC105206.3, AL591506.1, AP000864.1, AC022613.1, AC016999.1, and AL590428.1. Using these lncRNAs, a risk model was constructed. The AUCs for OS prediction after one, three, and five years were all above 0.7, demonstrating the remarkable prediction potential of this risk signature. Furthermore, the signature demonstrated a strong independent prognostic ability, and the nomogram combining the risk score and clinical features further improved prognosis prediction in LUAD.

The heatmap depicting the correlation between ICD genes and lncRNAs indicates that the selected lncRNAs are linked to key genes such as *BAX*, *NLRP3*, and *CASP1*, which are potential PD-1 inhibitors aiding immunotherapy. AC026355.2, a novel immune-related molecule involved in the immune response [19], autophagy [20], and necroptotic process in LUAD [21], is a crucial element that influences the development and prognosis of LUAD. *AC026355.2* expression was significantly positively correlated with *PIK3CA*, *TLR4*, *NLRP3*, *ENTPD1*, *IL1R1*, *IFNG*, and *EIF2AK3*, and negatively correlated with *CALR* and *BAX* expression (*p* < 0.001, Fig. 3E). PIK3CA enhances PI3K signaling and promotes tumor cell proliferation [22]. NLRP3 inflammasome activation contributes to inflammation and cancer development and mediates pyroptosis in various diseases [23]. IFNG, a cytokine interferon-gamma, is known to mediate cancer immunoevasion [24]. CALR has been associated with malignant transformation, tumor progression, and response to cancer therapy [25]. As a pro-apoptotic protein, BAX is involved in tumor progression and drug resistance [26]. Therefore, we hypothesize that AC026355.2, despite being rarely reported, is essential for tumor growth, and further research is necessary to determine its exact function. *LY86-AS1* is a lncRNA involved in the progression of multiple myeloma [27], Alzheimer’s disease [28], intracerebral hemorrhage [29], and LUAD [30]. According to our results, *LY86-AS1* expression was downregulated in LUAD cells, and patients with low LY86-AS1 expression had poorer OS than high-LY86-AS1 patients. Moreover, a high level of LY86-AS1 was correlated with high immune scores, indicating increased immune components in patients with high LY86-AS1 expression that possibly contributes to antitumor capacity. By enhancing our mechanistic understanding of LUAD, the newly acquired information on ICD-related lncRNAs may help bring a breakthrough in therapeutic practice.

Fewer immunogenic components can lead to tumor cells escaping antitumor immunotherapy [31]. The high- and low-risk groups in the risk model displayed distinct TME and TICs. Survival analysis demonstrated that the high-risk group had worse OS based on multiple clinical features. Conversely, the low-risk group had increased immune scores and more immune infiltration, including B cell infiltration. B cells have the potential to restrain tumor cells and reduce the occurrence of metastases, thereby limiting further tumor spread [32], which may contribute to better OS in individuals with a low risk. In NSCLC, M2 macrophage infiltration into tumor islets leads to a poor prognosis [33]. In this study, we found a positive correlation between the risk score and the proportion of M2 macrophages, which may result in poor OS in patients at a high risk. Furthermore, CIBERSORT analysis revealed reduced levels of memory B cells and increased M0 macrophage infiltration in high-risk patients. Liu et al. discovered that tumors deficient in memory B cells or with elevated M0 macrophages were related to a poor prognosis of LUAD at an earlier clinical stage [34], which is consistent with our results.

We evaluated the capability of the risk model, based on TIDE scores, to forecast immune evasion in the two risk groups and to further analyze its immunotherapeutic application. The results showed that the high-risk group exhibited decreased TIDE scores, indicating a higher possibility of benefiting from ICI treatment, and more gene expression of immune checkpoints. These findings suggest that our signature may serve as a valuable tool for evaluating the efficacy of immunotherapy in LUAD patients. In addition to immunotherapy, we also found that a patient’s risk profile could predict their response to immunotherapy, and high-risk patients responded well to first-line therapeutic drugs for NSCLC, including doxorubicin, paclitaxel, etoposide, cisplatin, and gemcitabine [4,35–39]. These results indicate that chemotherapy and targeted drugs have a significant impact on high-risk individuals, highlighting the importance of personalized anticancer therapy. GSEA revealed that the high-risk group exhibited significantly higher activation of the cell cycle, focal adhesion, ECM-receptor interaction, and spliceosome pathways than the low-risk group. The interactions between the ECM and cellular receptors are known to contribute to tumor growth and metastasis [40]. Consistent with DEGs enrichment analysis, medications that primarily target the cell cycle and DNA replication were effective in high-risk patients, suggesting their anticancer mechanisms. In contrast, erlotinib, which inhibits the epidermal growth factor receptor essential in cancer proliferation [41], was found to be more responsive in the low-risk category. Additionally, improved immunotherapy efficacy in the high-risk group may have contributed to patients’ successful treatment. These results provide potential therapeutic options for LUAD patients and could impact customized antitumor therapy.

The investigation yielded the following main results. First, a 10 ICD-related risk signature was developed and thoroughly investigated to predict the outcome of LUAD patients. Second, the risk score was found to be associated with clinicopathological traits and immune infiltration modification, identifying targets for future therapy. Third, sensitive drug prediction might be a promising treatment approach for improving LUAD immunotherapy and offering customized consequences for individualized treatment. Despite these encouraging results, this study had a few limitations. First, further research is required to better understand how genes are expressed and play a predictive function in the risk model at the protein level. Second, the tumor-immune milieu varies geographically, and the potential genes included in our study were limited to ICD marker genes, restricting the risk signature’s capacity for prognostic prediction. Finally, our study only included a description of mechanical analysis; therefore, additional research utilizing cellular and animal models is required to support our findings.

## Conclusions

ICD-related lncRNA risk signature could be useful for predicting outcome and developing effective treatment strategies for LUAD patients. Targeting ICD and ICD-related lncRNAs could be a viable approach to overcome multisystemic treatment failures and expand immunotherapy. This signature may serve as a prognostic biomarker for personalized disease outcome prediction and assist in selecting the most effective anticancer treatment.

## Supplementary Materials

**SUPPLEMENTARY FIGURE 1:**
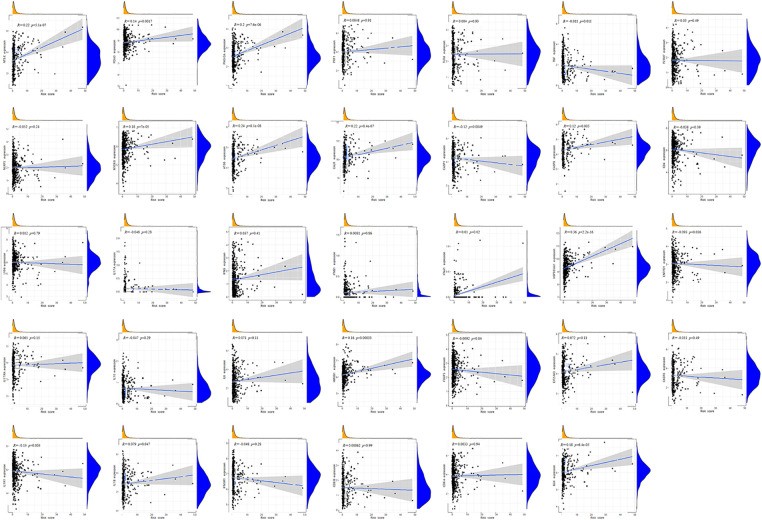
Correlations between ICD genes and risk scores.

## Data Availability

The datasets used and/or analyzed in the current study are available from the corresponding author on reasonable request.
